# Depth Sensor-Based Instrumentation of the Fukuda Stepping Test: Reliability and Clinical Associations in Older Adults

**DOI:** 10.3390/s26051623

**Published:** 2026-03-05

**Authors:** Hasan Tolga Ünal, Mertcan Koçak, Sebahat Yaprak Çetin, Özgün Kaya Kara, Mert Doğan

**Affiliations:** 1 Department of Physiotherapy and Rehabilitation, Faculty of Health Sciences, Akdeniz University, Antalya 07070, Türkiye; 2 Department of Mechatronics Engineering, Faculty of Engineering and Architecture, İzmir Kâtip Çelebi University, İzmir 35620, Türkiye

**Keywords:** Fukuda stepping test, depth sensor, markerless motion capture, postural control, balance assessment, older adults, reliability, kinematic analysis, Kinect v2

## Abstract

**Highlights:**

**What are the main findings?**

- Depth sensor-based instrumentation of the Fukuda Stepping Test demonstrated moderate-to-good test–retest reliability for most segmental kinematic parameters in older adults.
- Trunk flexion and rotational kinematic parameters showed clinically meaningful associations with cognitive function, physical activity, balance performance, and quality of life.

**What are the implications of the main findings?**

- Markerless depth-sensing technology provides objective and clinically relevant information beyond conventional Fukuda Stepping Test outcomes.
- Segmental kinematic parameters, particularly trunk flexion, may serve as practical indicators for multidomain functional assessment and fall-risk screening in older adults.

**Abstract:**

This study evaluated the test–retest reliability of a depth sensor-based Fukuda Stepping Test and examined associations between sensor-derived kinematic parameters and established clinical outcomes in older adults. Eighty-six community-dwelling older adults (mean age 70.3 ± 4.7 years) performed an eyes-closed stepping task monitored by a Microsoft Kinect v2 sensor. Clinical assessments included the Berg Balance Scale, Timed Up and Go test, Five Times Sit-to-Stand, Montreal Cognitive Assessment, International Physical Activity Questionnaire, and WHOQOL-OLD. Test–retest reliability was assessed using intraclass correlation coefficients in a randomly selected subgroup. Reliability estimates varied across parameters, with temporal and displacement-based measures demonstrating more consistent agreement across sessions, whereas selected angular variables showed greater variability. Correlation analyses identified statistically significant associations between trunk kinematic changes and clinical measures, with effect sizes generally ranging from weak to moderate magnitude. Upper trunk rotation was associated with functional mobility measures, while traditional displacement-based metrics demonstrated limited clinical relationships. These findings support the feasibility of markerless depth-sensing technology for objective quantification of movement during the Fukuda Stepping Test and highlight the potential contribution of segmental kinematic parameters to multidimensional functional assessment in older adults.

## 1. Introduction

Falls constitute a major public health concern among older adults, leading to increased morbidity, mortality, and healthcare costs. The annual prevalence of falls among community-dwelling older individuals has been reported to be 26.5%, and more than 14 million adults aged 65 years and older experience a fall each year in the United States alone [1,2]. Approximately 10–20% of falls result in fractures, and falls are estimated to have contributed to 684,000 deaths globally among older adults in 2017 [2,3]. Although exercise-based interventions are effective in reducing fall incidence [2], early and objective identification of individuals at increased fall risk remains a key challenge in clinical practice.

Age-related deterioration in vestibular, proprioceptive, and visual systems adversely affects postural control and increases susceptibility to falls [4,5]. Visual impairment has been associated with a 16% higher fall rate, rising to nearly 40% in individuals with blindness, while vestibular dysfunction has been shown to exert moderate negative effects on postural control parameters [5,6]. These sensory declines, often coexisting in older age, compromise multisensory integration and contribute to balance instability. Accordingly, clinical assessments that challenge sensory integration—especially by limiting visual feedback—play a critical role in evaluating postural control in older adults. The Fukuda Stepping Test (FST) is a simple and widely used assessment in which individuals step in place with their eyes closed while trunk rotation and linear displacement are observed relative to the starting position [7,8]. The test has been applied in vestibular and balance assessments, including studies involving older adults [7,8]. However, conventional implementations of the FST rely largely on subjective visual estimation or manual measurements, lack standardized protocols, and provide limited kinematic information. Reported test–retest reliability is moderate, and substantial variability exists across protocols [7,8]. Moreover, traditional FST outcomes are typically restricted to rotation and displacement measures, without capturing detailed three-dimensional kinematic behavior of the head and trunk [8].

The integration of motion-sensing technologies into traditional clinical balance tests reflects a broader transition from observational scoring toward quantitative movement analysis.

Depth-sensing technologies, such as Microsoft Kinect and Azure Kinect, enable markerless, relatively low-cost motion capture and provide automated access to three-dimensional kinematic data. These systems have been shown to be feasible and reliable for evaluating functional tasks, stepping, and balance in older adults in both laboratory and home-based settings [9,10]. Markerless depth sensors reduce preparation time, facilitate automation, and allow quantification of anterior–posterior, mediolateral, and vertical displacements, as well as head and trunk angular movements [9,10,11]. Previous studies have demonstrated meaningful associations between depth sensor-derived kinematic parameters and conventional clinical balance and mobility measures [9,10], and recent systematic reviews have reported good-to-excellent reliability of markerless camera-based systems compared with marker-based motion capture in gait and balance assessments [11].

Despite these advances, direct studies modernizing the FST using depth-sensing technology remain limited. While the reliability and protocol characteristics of the conventional FST in older adults have been explored [7,8], depth sensor-based implementations aligned specifically with the classical FST framework are scarce. Conversely, although Kinect-based stepping and balance tasks provide rich kinematic data, they have generally not been designed as standardized adaptations of the FST [9,10,11]. Importantly, recent inertial measurement unit-based analyses of the Fukuda–Unterberger test have demonstrated the feasibility of objective sensor-based quantification, reporting moderate test–retest reliability for stepping-on-the-spot paradigms [12]. However, these approaches primarily focused on rotational dynamics and did not examine multidomain clinical associations. This gap highlights the need for a standardized, sensor-based version of the FST with clearly defined protocols and objective outcome measures. Therefore, the aim of this study was to evaluate the test–retest reliability of a depth sensor-based, modernized FST in older adults and to examine the associations between sensor-derived kinematic parameters and established clinical measures of balance, mobility, fear of falling, and quality of life. The present study contributes to the literature by (1) proposing a standardized depth sensor-based adaptation of the Fukuda Stepping Test, (2) evaluating its test–retest reliability using a structured statistical framework, and (3) examining its associations with multidomain clinical outcomes in older adults.

## 2. Materials and Methods

### 2.1. Study Design and Participants

This cross-sectional methodological study was approved by the Akdeniz University Non-Interventional Clinical Research Ethics Committee (30.01.2025, No: TBAEK-168). All participants provided written informed consent according to the Declaration of Helsinki. The study was conducted between January and November 2025 at the Department of Physiotherapy and Rehabilitation, Faculty of Health Sciences, Akdeniz University, Antalya, Türkiye.

**Figure 1:**
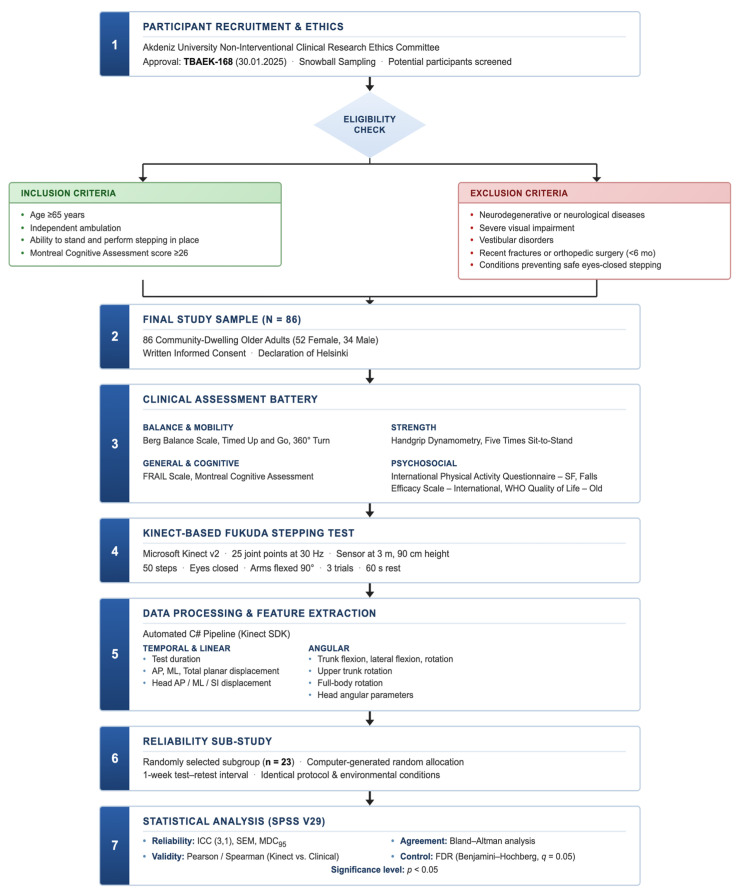
Research workflow showing participant selection, assessment procedures, testing protocol, data processing, and statistical analysis stages.

A total of 86 community-dwelling older adults (52 females, 34 males) were recruited using snowball sampling. Inclusion criteria were age ≥65 years, independent ambulation, ability to stand and perform stepping in place, and Montreal Cognitive Assessment (MoCA) score ≥26 to exclude cognitive impairment. Exclusion criteria included self-reported physician-diagnosed neurodegenerative disorders (e.g., Parkinson’s disease, atypical parkinsonism, cerebellar ataxia, or dementia), as these conditions are characterized by disease-specific impairments in axial control, gait initiation, and rotational stability that could directly influence trunk and head kinematics during the Fukuda Stepping Test. Other focal neurological conditions such as prior stroke, peripheral neuropathy, or multiple sclerosis were excluded due to their potential to produce asymmetric stepping patterns, altered proprioception, or impaired motor coordination. Clinically diagnosed vestibular disorders (e.g., benign paroxysmal positional vertigo, unilateral vestibular hypofunction, Ménière’s disease) were excluded because the FST is specifically sensitive to vestibular asymmetry and spatial orientation deficits, which would confound interpretation of age-related balance mechanisms. Severe visual impairment not adequately corrected with lenses (e.g., advanced macular degeneration or glaucoma) was excluded, as visual system deficits may alter baseline postural alignment and sensory reweighting even prior to eye closure. Recent lower-extremity fractures, joint replacement surgery, or major orthopedic procedures within six months were excluded to avoid compensatory stepping strategies, reduced weight-bearing symmetry, or incomplete biomechanical recovery affecting rotational displacement outcomes. Additionally, individuals with uncontrolled cardiovascular or systemic conditions that could compromise safe performance of repeated eyes-closed stepping were not included. Eligibility was determined through structured health screening interviews, participant medical history self-report, and cognitive testing prior to data collection. The retest subgroup (*n* = 23) was randomly selected from the full cohort using a simple random sampling procedure (computer-generated random allocation), thereby reducing selection bias and supporting representativeness of the reliability sample. The research workflow is shown in Figure 1.

### 2.2. Depth Sensor-Based Fukuda Stepping Test

Three-dimensional kinematic data were collected using Microsoft Kinect v2 (Microsoft Corp., Redmond, WA, USA), a time-of-flight depth sensor tracking 25 anatomical joint points at 30 Hz. The sensor was positioned 3 m in front of participants at 90 cm height to ensure optimal skeletal tracking and minimize occlusion. Raw joint coordinate data were recorded in real time using custom-developed C# language utilizing the Kinect Software Development Kit version 2.0 (Microsoft Corp., Redmond, WA, USA), enabling synchronized recording, trial labeling, and structured data storage. Kinect provides acceptable validity and reliability for postural control and balance assessment [13,14].

#### 2.2.1. Test Protocol

**Figure 2:**
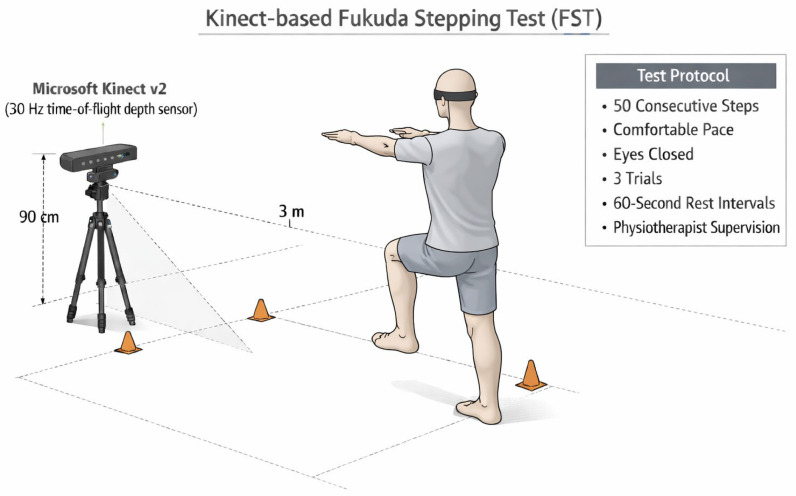
Kinect-based Fukuda Stepping Test.

Participants stood in the center of the capture area with arms flexed to approximately 90° in front of the body. They were instructed to close their eyes and perform 50 consecutive steps in place at a self-selected comfortable pace without intentionally correcting body orientation or step direction. The selection of 50 steps was based on previous methodological evidence indicating that a 50-step protocol provides superior test–retest reliability compared with longer step counts while minimizing fatigue-related variability [8]. Verbal instructions were standardized, and no feedback was given during execution. Each participant completed three trials with 60 s rest intervals between trials; mean values were used for analysis. A physiotherapist supervised the test, which was terminated prematurely if balance was lost, eyes were opened, or external support was required (Figure 2).

For test–retest reliability assessment, the protocol was repeated one week later in a subgroup of 23 participants under identical conditions. Traditional visual estimation was replaced by continuous three-dimensional kinematic recording, enabling objective quantification of trunk and head movements throughout the test. The same processing pipeline was applied for initial assessment and retest session.

#### 2.2.2. Data Extraction and Validation

Raw three-dimensional skeletal data were processed using custom-developed C# software that automatically extracted temporal, linear displacement, and angular kinematic parameters from joint coordinates defined within a global reference frame. The cut-off frequency was selected in accordance with the system’s sampling rate of 30 Hz and the low-frequency characteristics of slow stepping and postural movements.

Skeletal tracking outputs were visually verified against synchronized video recordings to identify potential tracking artifacts, including transient deviations related to occlusion. Derived kinematic parameters were additionally examined against physiologically plausible anatomical range-of-motion limits to detect potential outliers. Verification procedures were limited to artifact identification and, where necessary, exclusion of affected segments; no manual editing of coordinate trajectories was performed.

Extracted parameters were categorized as follows:

Temporal parameter: Test duration defined as the time elapsed between the first and last detected step.

Linear displacement parameters: Trunk anterior–posterior and medio-lateral displacement relative to the initial position. Total planar displacement calculated as horizontal Euclidean distance. Head displacement in anterior–posterior, medio-lateral, and superior–inferior directions.

Angular parameters: Trunk flexion–extension, lateral flexion, and axial rotation relative to global coordinates. Upper trunk rotation and full-body rotation based on relative orientation between upper-body and lower-extremity segments. Head flexion–extension, lateral flexion, and axial rotation relative to the neck segment (Supplementary Materials File S2).

All parameters were calculated separately for each trial, and mean values across three trials were used for statistical analysis.

### 2.3. Clinical Outcomes

All clinical assessments were administered prior to the sensor-based FST in a standardized sequence by same physiotherapist. Tests were conducted in a controlled indoor environment under consistent lighting and safety conditions. Standardized verbal instructions were provided for each assessment according to validated administration guidelines, and no additional feedback was given during performance. Rest intervals were allowed when necessary to prevent fatigue-related performance bias. Assessments were selected to characterize balance performance, functional mobility, muscle strength, frailty status, cognitive function, physical activity level, fear of falling, and quality of life—domains commonly associated with postural control and fall risk in older adults.

#### 2.3.1. Balance Assessment

Balance performance was evaluated using the Berg Balance Scale (BBS). The BBS consists of 14 items assessing static and dynamic balance during functional tasks (e.g., standing, transfers, turning, reaching). Each item is scored from 0 to 4, yielding a total score ranging from 0 to 56, with higher scores indicating better balance performance [15].

#### 2.3.2. Functional Mobility

Functional mobility was assessed using the Timed Up and Go (TUG) test. Participants rose from a chair, walked 3 m at a comfortable pace, turned, returned to the chair, and sat down. Total time was recorded in seconds, with longer times indicating poorer functional mobility and dynamic balance [16].

#### 2.3.3. Dynamic Turning Ability

Dynamic turning ability was evaluated using the 360° Turn Test. Participants completed a full 360-degree turn, and both turning time and number of steps were recorded. Turning performance is an important component of dynamic balance and has been associated with fall risk in older adults [17].

#### 2.3.4. Upper Extremity Muscle Strength

Upper extremity muscle strength was assessed using handgrip strength, measured with a hand dynamometer (Fabrication Enterprises Inc., White Plains, NY, USA) and recorded in kilograms. Handgrip strength is commonly used as a proxy for overall muscle strength and has been associated with functional performance and adverse health outcomes in older adults [18].

#### 2.3.5. Lower-Extremity Functional Strength

Lower-extremity functional strength was evaluated using the Five Times Sit-to-Stand Test (5xSTS). Participants stood up from and sat down on a standard chair five consecutive times as quickly as possible without using their arms. Total time was recorded in seconds, with longer times indicating reduced lower limb strength and functional capacity [19].

#### 2.3.6. Frailty

Frailty status was assessed using the FRAIL scale, a brief screening tool consisting of five components: fatigue, resistance, ambulation, illnesses, and weight loss. Each component is scored dichotomously, yielding a total score from 0 to 5, with higher scores indicating greater frailty [20].

#### 2.3.7. Cognitive Performance

Global cognitive function was screened using the Montreal Cognitive Assessment (MoCA). The MoCA assesses multiple cognitive domains, including attention, executive functions, memory, language, visuospatial abilities, and orientation, with a maximum score of 30 [21].

#### 2.3.8. Physical Activity

Physical activity level was assessed using the International Physical Activity Questionnaire–Short Form (IPAQ-SF), which captures self-reported physical activity over the previous seven days across different intensity levels. Results were expressed as metabolic equivalent task minutes per week (MET-min/week), providing an estimate of overall physical activity [22,23].

#### 2.3.9. Fear of Falling

Fear of falling was evaluated using the Falls Efficacy Scale–International (FES-I), which assesses concern about falling during a range of daily activities. Higher scores indicate greater fear of falling [24].

#### 2.3.10. Quality of Life

Quality of life was assessed using the World Health Organization Quality of Life Instrument–Older Adults Module (WHOQOL-OLD). This instrument evaluates domains particularly relevant to aging, including sensory abilities, autonomy, social participation, and intimacy. Higher scores reflect better perceived quality of life [25].

### 2.4. Statistical Analysis

All analyses were performed using IBM SPSS Statistics version 29.0 (IBM Corp., Armonk, NY, USA). Descriptive statistics were calculated for all variables; continuous variables were summarized as mean ± standard deviation, categorical variables as frequencies and percentages. Distribution was examined using histograms and Shapiro–Wilk test.

Test–retest reliability was assessed using intraclass correlation coefficient (ICC) based on a two-way mixed-effects model with absolute agreement (ICC [3,1]). ICC values were interpreted according to Koo and Li as poor (<0.50), moderate (0.50–0.75), good (0.75–0.90), or excellent (>0.90) [26]. Measurement precision was quantified by standard error of measurement (SEM = SD × √(1 − ICC)) and minimal detectable change at 95% confidence level (MDC_95_ = SEM × 1.96 × √2). Also, 95% confidence intervals were calculated for ICC estimates to provide precision bounds consistent with established reliability reporting standards. Agreement was examined using Bland–Altman analysis with mean differences and 95% limits of agreement (Supplementary Materials File S1).

Associations between sensor-derived kinematic parameters and clinical outcomes were analyzed using Pearson’s correlation coefficient for normally distributed variables and Spearman’s rank correlation coefficient otherwise. To control for Type I error across multiple comparisons, clinical outcomes were categorized into five a priori hypothesized domains: (1) Balance and Mobility, (2) Strength, (3) Physical Activity, (4) Cognitive Function, and (5) Quality of Life/Frailty. Within each domain, we hypothesized that specific depth sensor–derived kinematic parameters would show significant clinical associations. The statistical significance of correlations within these hypothesized domains was tested using the Benjamini–Hochberg false discovery rate (FDR) procedure to adjust *p*-values. Any correlations identified outside these predefined hypothesized structures were treated as exploratory and interpreted with caution. Correlation magnitudes were interpreted according to Evans as very weak (<0.20), weak (0.20–0.39), moderate (0.40–0.59), strong (0.60–0.79), or very strong (≥0.80) [27]. Statistical significance was set at *p* < 0.05.

## 3. Results

A total of 86 community-dwelling older adults were included in the analysis. The cohort had a mean age of 70.3 ± 4.7 years and a mean body mass index of 27.52 ± 3.61 kg/m^2^; 60.5% were female. Comprehensive demographic and clinical characteristics are summarized in Table 1.

Descriptive statistics for all sensor-derived kinematic parameters obtained during the test and retest sessions are presented in Table 2.

Test duration and linear displacement measures were lower at retest compared with the initial assessment. Anterior–posterior displacement represented the largest linear component in both sessions, whereas medio-lateral displacement remained smaller. Total planar displacement followed a similar pattern.

Among angular parameters, upper trunk rotation and full-body rotation exhibited the greatest angular deviations in both sessions. Head axial rotation showed the highest variability among head kinematic measures (Table 2). Test–retest reliability and post hoc power results for the depth sensor-based Fukuda Stepping Test parameters are summarized in Table 3.

Test duration and anterior–posterior displacement demonstrated similar correlation and intraclass correlation coefficients, with confidence intervals remaining within positive ranges. Total planar displacement and head anterior–posterior displacement showed comparable levels of agreement. Medio-lateral trunk displacement exhibited lower correlation and wider confidence intervals. Among angular measures, trunk flexion change and full-body rotation change showed moderate ICC values, whereas trunk lateral flexion and upper trunk rotation demonstrated low agreement with confidence intervals crossing zero.

Head kinematic parameters displayed heterogeneous results. Head lateral flexion showed relatively higher agreement, while head flexion–extension, axial rotation, and selected displacement measures presented lower ICC values and broader confidence intervals. SEM and MDC values varied substantially across parameters, with greater measurement error observed in selected angular variables (Table 3).

Summary of clinical assessments, physical performance, and quality of life outcomes of the participants are presented in Table 4.

Handgrip strength and Five Times Sit-to-Stand performance averaged at 28.76 ± 8.01 kg and 11.72 ± 2.20 s, respectively. Mobility and balance assessments yielded mean values of 10.55 ± 1.93 s for TUG, 2.99 ± 0.55 s for 360° turning time, and 52.20 ± 2.60 for the Berg Balance Scale. Frailty and cognitive assessments showed mean scores of 0.30 ± 0.60 and 26.70 ± 0.70, respectively. The mean Falls Efficacy Scale–International score was 23.20 ± 4.03. Total physical activity averaged 1373.16 ± 756.01 MET-min/week. The WHOQOL-OLD total score was 75.31 ± 11.04, with domain-specific values detailed in Table 4.

Correlation analyses between depth sensor-based Fukuda Stepping Test parameters and muscle strength, frailty status, cognitive function, physical activity level are presented in Table 5. 

Correlation analyses between depth sensor-based Fukuda Stepping Test parameters and balance, functional mobility and quality of life outcomes are presented in Table 6. 

Correlation analyses revealed several statistically significant associations between depth sensor-based FST parameters and clinical assessment outcomes. Trunk flexion change demonstrated moderate negative correlations with cognitive performance and total physical activity. Weak-to-moderate negative associations were observed between trunk flexion change and quality of life, while a weak negative correlation was identified with balance performance.

Upper trunk rotation change showed moderate negative correlations with functional lower-extremity performance and TUG duration. Full-body rotation change exhibited a weak positive correlation with total physical activity.

Head kinematic parameters were also associated with selected clinical measures. Head mediolateral displacement showed weak positive correlations with frailty, lower-extremity performance, and fear of falling. Head superior–inferior displacement demonstrated weak-to-moderate positive associations with physical activity and weak positive correlations with quality of life. Head flexion change showed a weak negative correlation with daily walking duration. No statistically significant correlations were observed between test duration, anterior–posterior displacement, or total planar displacement and most clinical outcomes (*p* < 0.05) (Table 5 and Table 6).

## 4. Discussion

This study evaluated the test–retest reliability of a depth sensor-based FST and examined correlations between sensor-derived kinematic parameters and clinical outcomes in community-dwelling older adults. The Microsoft Kinect v2-based protocol yielded poor-to-moderate reliability for temporal and several kinematic parameters, with angular parameters exhibiting superior repeatability compared to linear displacement measures. Trunk flexion emerged as a multidimensional indicator, showing weak-to-moderate negative associations with cognitive function, physical activity, balance performance, and quality of life. Upper trunk rotation correlated negatively with functional mobility, while head superior–inferior displacement showed positive associations with physical activity and quality of life. Notably, traditional global displacement metrics showed weak or non-significant associations with clinical outcomes, suggesting that markerless depth sensor technology captures clinically meaningful segmental kinematic patterns that substantially exceed conventional FST metrics.

Recent FST modernization efforts have primarily employed wearable inertial measurement units (IMUs). Belluscio et al. developed the instrumented FST using five IMU sensors, demonstrating that upper-body stability indices discriminated between motor deficit levels in stroke patients despite non-significant differences in conventional displacement measures [28]. Hemm et al. employed nine Xsens IMU sensors, reporting excellent correlation between IMU-derived and manual angular measurements with moderate test–retest reliability for global displacement but emphasized that dynamic parameters provided additional discriminatory value [12]. Miwa et al. (2023) used three motion sensors at head, thorax, and lumbar spine, demonstrating that segmental motion increased substantially under eyes-closed conditions [29].

The present depth sensor approach offers distinct advantages: markerless capture eliminates donning/doffing procedures and sensor placement variability, avoids cumulative IMU drift error, and provides direct skeletal tracking without complex sensor fusion algorithms. However, single-camera configurations are vulnerable to occlusion artifacts, whereas body-worn IMUs maintain contact throughout motion sequences. Critically, both IMU-based and depth sensor studies converge on a key finding: segmental kinematic parameters provide superior clinical information compared to traditional global displacement metrics.

The poor-to-moderate reliability observed for angular kinematic parameters aligns with recent findings supporting markerless motion capture. Guess et al. reported that Azure Kinect-based gait analyses demonstrated high agreement with gold-standard optical motion capture systems, with Pearson correlation coefficients exceeding 0.87 for spatiotemporal parameters such as step length and step width [30]. Similarly, Albert et al. showed that Azure Kinect provides significantly higher accuracy in spatial gait parameters compared with previous-generation sensors, with notable improvements in foot tracking performance [31]. In addition, Bertrand et al. reported excellent agreement (ICC > 0.90) between tablet-based markerless motion capture applications and expert clinician ratings during the 30-Second Sit-to-Stand and Timed Up and Go tests, supporting the clinical validity of this technology [32]. However, as noted by Lim et al. while flexion–extension movements of lower extremity joints exhibit high reliability, movements in the abduction–adduction plane demonstrate lower reliability [33]. The lower reliability observed in selected parameters may reflect both technical and biological sources of variability, including single-camera occlusion, skeletal tracking limitations, and intra-individual motor variability during repeated stepping. Differences across studies may also arise from variations in measurement methodology and sample characteristics. As previous reports do not consistently provide detailed clinical profiles of study populations, direct comparison of reliability estimates remains limited. The present study contributes by combining transparent kinematic processing with clearly described clinical characteristics, thereby supporting more interpretable integration of technological and clinical assessments in future research. Also, the relatively small retest subsample may have influenced the stability of certain reliability estimates. It is well established that intraclass correlation coefficients can be sensitive to sample size and data imbalance, particularly in movement analysis research. As discussed by Trabassi et al., small-sample designs may limit the robustness and generalizability of classification and reliability outcomes in sensor-based assessments [34]. Therefore, while the present findings provide preliminary reliability evidence, future multicenter studies including larger and more heterogeneous samples are warranted to further strengthen the stability and external validity of depth sensor-based FST implementations.

The moderate negative correlation between trunk flexion and cognitive performance indicates that individuals with lower cognitive capacity exhibit greater trunk flexion during eyes-closed stepping, reflecting impaired cognitive–motor integration or reduced executive control over posture. In line with the cognitive penetrability hypothesis proposed by Boisgontier et al., postural control in aging shifts away from automatic regulation toward increased reliance on cortical and voluntary control mechanisms, thereby heightening dependence on general cognitive resources to maintain balance [35]. Marusic et al. further emphasized that increased activation of the dorsolateral prefrontal cortex, particularly during challenging tasks, reflects this elevated cognitive load and highlights the critical role of executive function capacity in shaping postural strategies [36]. Within this framework, when executive resources become insufficient, the postural control system may revert to more primitive or seemingly safer postural configurations. Excessive trunk flexion may therefore represent a compensatory strategy adopted by individuals with reduced processing speed, whereby stability is enhanced through lowering the center of mass at the expense of postural efficiency. The weaker association observed between trunk flexion and balance performance compared with cognitive performance suggests that cognitive–motor integration may be a stronger determinant of trunk kinematic behavior than balance capacity alone.

Trunk flexion showed consistent negative associations with physical activity, balance performance, and quality of life in the present study, suggesting a potential relationship between sagittal-plane trunk kinematics and multidomain functional status. Previous biomechanical research has reported associations between sagittal-plane trunk kinematics and balance recovery outcomes following destabilizing perturbations, indicating that trunk motion may be relevant in fall-related contexts [37]. In addition, sensor-based fall-risk assessment studies have reported that trunk-related kinematic features are associated with frailty and fall-risk indicators beyond global performance measures alone [38]. In contrast, the absence of significant associations for trunk lateral flexion suggests that, within the present eyes-closed stepping paradigm, sagittal-plane trunk motion was more closely related to the selected clinical measures than frontal-plane trunk motion. Given the cross-sectional design, these findings should be interpreted as associative rather than mechanistic.

The negative associations observed between upper trunk rotation and functional mobility measures (5xSTS and TUG) suggest that excessive axial rotation during eyes-closed stepping may reflect underlying limitations in transfer- and turning-related mobility. This interpretation is consistent with evidence from instrumented TUG studies demonstrating that trunk kinematic features, particularly during transitional and turning phases, provide clinically relevant information beyond total task duration alone [39]. In addition, the positive associations between head mediolateral displacement and frailty, 5xSTS performance, and fear of falling suggest that increased lateral head motion reflects compromised mediolateral stability and reduced balance confidence. Supporting this interpretation, recent evidence indicates that fear of falling in older adults is associated with impaired mediolateral postural control and altered balance strategies [40]. Furthermore, the positive relationships observed between head superior–inferior displacement and both physical activity level and quality of life may reflect greater stepping amplitude and more dynamic movement execution in physically active individuals, whereas reduced vertical head motion may indicate more constrained and cautious movement strategies in less confident or less active individuals.

Traditional FSTs typically rely on subjective tools such as protractors and tape measures to quantify performance. However, research indicates that these global metrics often show weak associations with specific underlying impairments, whereas segmental kinematics provide robust insights into functional control. Global measures (e.g., distance or duration) reflect cumulative endpoints that can be achieved through diverse compensatory strategies, thereby obscuring specific neuromuscular control deficits [41,42]. In contrast, kinematic analysis reveals the subtle mechanisms of movement invisible to traditional scoring. This limitation underscores the need for modernizing clinical balance assessments to incorporate objective, instrumented measurement, a perspective strongly advocated in recent translational research [43]. In this perspective, depth sensor-based FST offers substantial clinical applicability. Trunk flexion’s associations with cognitive function, physical activity, balance, and quality of life suggest this parameter may serve as a pragmatic screening indicator for multidomain functional capacity and cognitive–motor integration deficits. Integration into digital health platforms could support remote fall-risk stratification, telerehabilitation monitoring, and early functional decline detection. Multiplanar kinematic data may facilitate individualized intervention targeting by identifying specific movement deficits warranting therapeutic attention.

Strengths include automated objective kinematic analysis, comprehensive clinical assessment battery, rigorous reliability evaluation, and markerless approach advantages (no donning/doffing, no drift error, simplified processing). However, single-sensor setup may introduce occlusion artifacts and precludes ambulatory assessment possible with IMU devices. The cross-sectional design precludes causal inference regarding whether trunk flexion causes or results from reduced cognitive/physical function. Sample characteristics (relatively high functional capacity, no diagnosed cognitive/neurological conditions) may limit generalizability to frailer populations. In addition, the high prevalence of self-reported fall history in the sample may have influenced postural strategies and movement variability, potentially affecting the magnitude of observed associations. Although this may limit generalizability to non-faller populations, it increases the ecological relevance of the findings for individuals at elevated fall risk. Also, given the cross-sectional design, the observed associations should be interpreted as relational findings rather than evidence of causal or mechanistic pathways. Weak-to-moderate correlation magnitudes (r = 0.22–0.39) suggest individual parameters have limited standalone predictive value, emphasizing need for multivariate modeling. The present findings should be interpreted within the context of a relatively high-functioning community-dwelling sample; therefore, clinically meaningful thresholds or predictive cut-off values cannot be derived from the current data. Future research should focus on validating these kinematic markers in frailer and clinically vulnerable populations and on establishing clinically interpretable reference values.

Longitudinal studies should determine whether trunk flexion patterns prospectively predict cognitive decline, falls, or functional deterioration, establishing directionality and potential causality. Machine learning integration could identify kinematic patterns associated with specific clinical phenotypes (sarcopenia, mild cognitive impairment, vestibular dysfunction). Validation of unsupervised home-based protocols using consumer-grade depth sensors could democratize objective balance screening. Domain-specific cognitive assessments could clarify which subdomains (executive function, visuospatial processing) most strongly drive trunk flexion associations. Comparative studies evaluating depth sensor versus IMU-based protocols regarding clinical utility, cost-efficiency, and user acceptability would inform technology selection. Intervention studies examining whether targeted rehabilitation modifies FST kinematics and associated outcomes would establish parameter responsiveness and therapeutic relevance.

## 5. Conclusions

This study demonstrates that depth sensor-based modernization of the Fukuda Stepping Test provides reliable, objective assessment of postural control in community-dwelling older adults, with segmental kinematic parameters, particularly trunk flexion, exhibiting clinically meaningful associations with cognitive function, physical activity, balance performance, and quality of life that substantially exceed traditional displacement-only metrics. The convergence of findings across multiple sensor modalities (depth sensors and IMUs) establishes that conventional FST scoring inadequately captures the biomechanical complexity of postural control, supporting the integration of affordable, markerless motion capture technology into geriatric assessment protocols. Trunk flexion emerges as a promising multidimensional marker warranting investigation as an early indicator of cognitive–motor integration deficits and a potential target for individualized rehabilitation interventions in aging populations.

## Figures and Tables

**Table 1:** Demographic and clinical characteristics of participants.

Variable	Total (n = 86)
**Age (years), mean ± SD**	70.3 ± 4.7
**Sex, n (%)**	
Female	52 (60.5)
Male	34 (39.5)
**Height (cm), mean ± SD**	163.6 ± 10.2
**Body weight (kg), mean ± SD**	73.7 ± 11.7
**Body mass index (kg/m^2^), mean ± SD**	27.52 ± 3.61
**Dominant side, n (%)**	
Right	80 (93.0)
Left	6 (7.0)
**History of falls, n (%)**	
Yes	71 (82.6)
No	15 (17.4)

**Table 2:** Test–retest descriptive statistics of the depth sensor-based Fukuda Stepping Test parameters.

Parameter	Test (n = 86) Mean ± SD	Retest (n = 23) Mean ± SD
Test duration (s)	236.92 ± 39.28	216.60 ± 32.48
AP displacement (cm)	−72.88 ± 29.85	−66.69 ± 23.57
ML displacement (cm)	−6.06 ± 30.31	1.10 ± 24.77
Total planar displacement (cm)	80.52 ± 29.28	72.80 ± 23.53
Trunk flexion change (°)	4.10 ± 5.21	2.57 ± 3.42
Trunk lateral flexion change (°)	−9.79 ± 7.67	−13.60 ± 8.59
Upper trunk rotation change (°)	83.54 ± 7.40	88.03 ± 17.64
Full-body rotation change (°)	83.73 ± 14.12	83.71 ± 23.18
Head flexion/extension change (°)	2.60 ± 10.22	1.83 ± 10.90
Head axial rotation change (°)	−45.09 ± 71.84	−31.49 ± 102.40
Head lateral flexion change (°)	3.25 ± 13.67	3.60 ± 15.96
Head ML displacement (cm)	−5.41 ± 29.50	1.90 ± 25.88
Head SI displacement (cm)	−5.47 ± 5.96	−2.64 ± 4.10
Head AP displacement (cm)	−74.77 ± 29.07	−68.28 ± 22.74

SD, standard deviation; AP, anterior–posterior; ML, mediolateral; SI, superior–inferior.

**Table 3:** Test–retest reliability of the depth sensor-based Fukuda Stepping Test parameters (n = 23).

Parameter (n = 23)	r	*p*	ICC (3,1)	95% CI	*p* (ICC)	Power	SEM	MDC^95^
Test duration (s)	0.65	<0.001	0.65	0.33–0.83	<0.001	0.96	23.24	64.41
AP displacement (cm)	0.67	<0.001	0.63	0.30–0.82	<0.001	0.98	18.16	50.33
ML displacement (cm)	0.35	0.10	0.35	−0.07–0.66	0.04	0.37	24.44	67.74
Total planar displacement (cm)	0.62	<0.001	0.58	0.23–0.80	0.001	0.93	18.98	52.60
Trunk flexion change (°)	0.47	0.03	0.46	0.07–0.73	0.01	0.64	3.83	10.61
Trunk lateral flexion change (°)	0.03	0.89	−0.20	−0.56–0.23	0.82	0.05	8.40	23.29
Upper trunk rotation change (°)	−0.03	0.88	0.01	−0.40–0.41	0.48	0.05	7.36	20.41
Full-body rotation change (°)	0.30	0.17	0.47	0.08–0.73	0.01	0.28	10.28	28.49
Head flexion/extension change (°)	0.19	0.38	0.29	−0.13–0.62	0.09	0.14	8.61	23.87
Head axial rotation change (°)	0.31	0.15	0.35	−0.06–0.66	0.04	0.30	57.92	160.54
Head lateral flexion change (°)	0.70	<0.001	0.68	0.38–0.85	<0.001	0.99	7.73	21.43
Head ML displacement (cm)	0.39	0.07	0.29	−0.13–0.62	0.08	0.46	24.86	68.90
Head SI displacement (cm)	0.38	0.07	0.38	−0.03–0.68	0.04	0.44	4.69	13.01
Head AP displacement (cm)	0.63	<0.001	0.60	0.25–0.81	0.001	0.94	18.39	50.96

AP, anterior–posterior; ML, mediolateral; SI, superior–inferior; n, sample size; r, correlation coefficient; *p*, significance level; ICC (3,1), intraclass correlation coefficient based on a two-way mixed-effects model for single measurements; 95% CI, 95% confidence interval; *p* (ICC), significance level of the ICC; Power, statistical power; SEM (Standard Error of Measurement), standard error of measurement; MDC95 (Minimal Detectable Change at the 95% confidence level), the smallest change that exceeds measurement error with 95% confidence.

**Table 4:** Summary of clinical assessments, physical performance, and quality-of-life outcomes (n = 86).

Clinical Measure	Mean ± SD	Min–Max
**Physical Performance and Strength**		
Handgrip strength (kg)	28.76 ± 8.01	15.36–49.43
5xSTS (s)	11.72 ± 2.20	6.33–16.20
**Mobility and Balance**		
360° Turning time (s)	2.99 ± 0.55	1.97–4.67
TUG (s)	10.55 ± 1.93	6.43–15.40
BBS (score)	52.20 ± 2.60	45–56
**Frailty and Cognitive Status**		
Frailty score	0.30 ± 0.60	0.00–2.00
MoCA (score)	26.70 ± 0.70	26.00–28.00
**Fear of Falling**		
Falls Efficacy Scale–International (FES-I), score	23.20 ± 4.03	15–34
Low fear, n (%)	15 (17.4)	—
Moderate fear, n (%)	58 (67.4)	—
High fear, n (%)	13 (15.1)	—
**Physical Activity (IPAQ)**		
Moderate-intensity activity (days/week)	2.40 ± 2.00	0.00–7.00
Moderate-intensity activity (hours/week)	0.64 ± 0.50	0.00–2.00
Walking (days/week)	5.20 ± 1.80	0.00–7.00
Walking (hours/week)	0.71 ± 0.30	0.00–1.50
Sitting time (hours/day)	7.30 ± 1.40	3.00–10.00
Total MET value (MET-min/week)	1373.16 ± 756.01	198.00–3096.00
Low activity, n (%)	11 (12.8)	—
Moderate activity, n (%)	72 (83.7)	—
High activity, n (%)	3 (3.5)	—
**Quality of Life (WHOQOL-OLD)**		
Sensory Abilities (SAB)	65.70 ± 25.03	6.25–100.00
Past, Present and Future Activities (PPF)	75.15 ± 10.89	43.75–100.00
Autonomy (AUT)	77.54 ± 17.19	6.25–100.00
Social Participation (SOP)	72.17 ± 15.33	18.75–100.00
Death and Dying (DAD)	86.05 ± 18.88	25.00–100.00
Intimacy (INT)	75.29 ± 16.66	31.25–100.00
WHOQOL-OLD total score	75.31 ± 11.04	34.38–94.7

5xSTS, Five Times Sit-to-Stand Test; BBS, Berg Balance Scale; FES-I, Falls Efficacy Scale–International; IPAQ, International Physical Activity Questionnaire; MET, metabolic equivalent of task; MoCA, Montreal Cognitive Assessment; SAB, Sensory Abilities; PPF, Past, Present and Future Activities; AUT, Autonomy; SOP, Social Participation; DAD, Death and Dying; INT, Intimacy; SD, standard deviation.

**Table 5:** Correlations between depth sensor-based Fukuda Stepping Test parameters and muscle strength, frailty status, cognitive function, physical activity level outcomes (n = 86).

FST Parameter	Handgrip (r)	5xSTS (r)	MoCA (r)	Frailty (r)	IPAQ (MET) (r)
Test duration (s)	−0.14	0.04	−0.01	−0.17	0.08
AP displacement (cm)	−0.03	0.06	−0.16	−0.09	0.07
ML displacement (cm)	0.14	0.07	0.001	0.01	0.01
Total planar displacement (cm)	−0.04	−0.06	0.10	−0.01	−0.04
Trunk flexion change (°)	−0.19	−0.09	−0.38 ** q	−0.10	−0.39 ** q
Trunk lateral flexion change (°)	0.22	−0.01	0.02	−0.15	−0.19
Upper trunk rotation change (°)	−0.08	−0.30 ** q	0.09	−0.20	−0.02
Full-body rotation change (°)	−0.11	0.12	0.01	0.01	0.24 * q
Head flexion change (°)	−0.04	−0.02	−0.06	−0.20	−0.15
Head axial rotation change (°)	−0.08	−0.17	0.16	−0.18	0.02
Head lateral flexion change (°)	−0.03	0.14	−0.02	0.13	0.01
Head ML displacement (cm)	0.14	0.22	−0.15	0.22 * q	0.01
Head SI displacement (cm)	0.11	0.04	−0.01	0.00	0.29 **
Head AP displacement (cm)	0.04	0.07	−0.10	0.01	0.07

Frailty, Frail Scale; 5xSTS, Five Times Sit-to-Stand Test; MoCA, Montreal Cognitive Assessment; IPAQ, International Physical Activity Questionnaire; MET, metabolic equivalent of task, * *p* < 0.05, ** *p* < 0.01 (two-tailed for exploratory analysis), q indicates Benjamini–Hochberg FDR-adjusted *p*-values within each clinical domain (q < 0.05 considered significant).

**Table 6:** Correlations between depth sensor-based Fukuda Stepping Test parameters and balance, functional mobility, and quality of life outcomes (n = 86).

FST Parameter	QoL	360° (r)	TUG (r)	BBS (r)	FES-I (r)
Test duration (s)	0.03	−0.08	0.12	0.21	−0.02
AP displacement (cm)	0.06	0.04	0.06	−0.12	0.03
ML displacement (cm)	0.06	0.14	0.23 * q	−0.16	0.28 * q
Total planar displacement (cm)	−0.05	−0.04	−0.06	0.10	−0.01
Trunk flexion change (°)	−0.29 ** q	−0.07	−0.04	−0.24 * q	0.05
Trunk lateral flexion change (°)	−0.001	0.22 *	−0.01	0.02	−0.15
Upper trunk rotation change (°)	0.05	−0.08	−0.30 ** q	0.09	−0.20
Full-body rotation change (°)	0.13	−0.11	0.12	0.01	0.01
Head flexion change (°)	−0.02	−0.04	−0.02	−0.06	−0.20
Head axial rotation change (°)	0.001	−0.08	−0.17	0.16	−0.18
Head lateral flexion change (°)	−0.01	−0.03	0.14	−0.02	0.13
Head ML displacement (cm)	0.04	0.14	0.22 * q	−0.15	0.22 *
Head SI displacement (cm)	0.26 *	0.11	0.04	−0.01	0.001
Head AP displacement (cm)	0.07	0.04	0.07	−0.10	0.01

QoL, WHOQOL-OLD; 360°, 360 Turn Test; TUG, Timed up and go test; BBS, Berg Balance Scale; FES-I, Falls Efficacy Scale–International; * *p* < 0.05, ** *p* < 0.01 (two-tailed for exploratory analysis), q indicates Benjamini–Hochberg FDR-adjusted *p*-values within each clinical domain (q < 0.05 considered significant).

## Data Availability

The data presented in this study are not publicly available due to ethical and privacy restrictions, as they contain information that could compromise the confidentiality of research participants. Data supporting the findings of this study are available from the corresponding author upon reasonable request and with permission of the relevant institutional ethics committee.

## Supplementary Materials

The following supporting information can be downloaded at https://www.mdpi.com/article/10.3390/s26051623/s1.

## Abbreviations

The following abbreviations are used in this manuscript:

FST	Fukuda Stepping Test
AP	Anterior–posterior
ML	Mediolateral
SI	Superior–inferior
ICC	Intraclass correlation coefficient
SEM	Standard error of measurement
MDC	Minimal detectable change
MDC95	Minimal detectable change at the 95% confidence level
SD	Standard deviation
MoCA	Montreal Cognitive Assessment
BBS	Berg Balance Scale
TUG	Timed Up and Go test
5xSTS	Five Times Sit-to-Stand Test
FES-I	Falls Efficacy Scale–International
IPAQ	International Physical Activity Questionnaire
IPAQ-SF	International Physical Activity Questionnaire–Short Form
MET	Metabolic equivalent of task
WHOQOL-OLD	World Health Organization Quality of Life Instrument–Older Adults Module
IMU	Inertial measurement unit
QoL	Quality of life
