# Supplementary material for: Depth Sensor-Based Instrumentation of the Fukuda Stepping Test: Reliability and Clinical Associations in Older Adults
Source: Sensors (Basel). 2026 Mar 5;26(5):1623. doi: 10.3390/s26051623 (PMC12986737; doi:10.3390/s26051623)
Supplement: Supplementary file 1 [file sensors-26-01623-s001.zip › Supplementary Material 1.pdf]

APPENDIX A

Bland–Altman plots were used to visualize the agreement between test and retest measurements and are presented below.

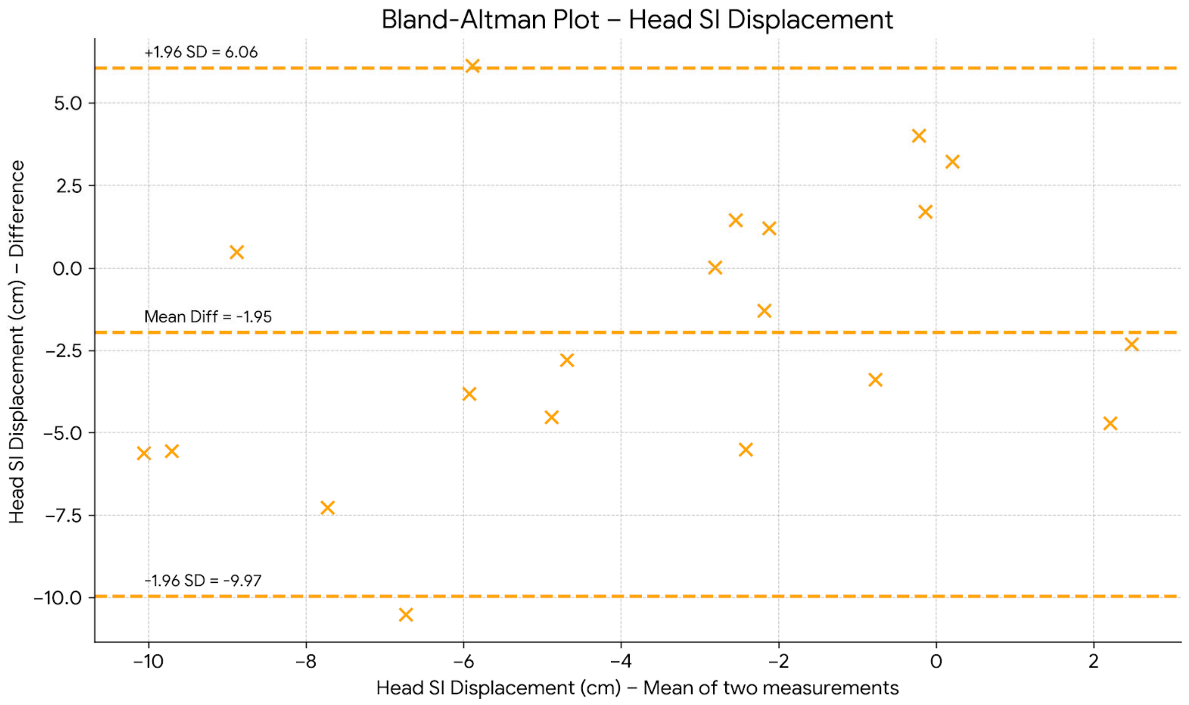

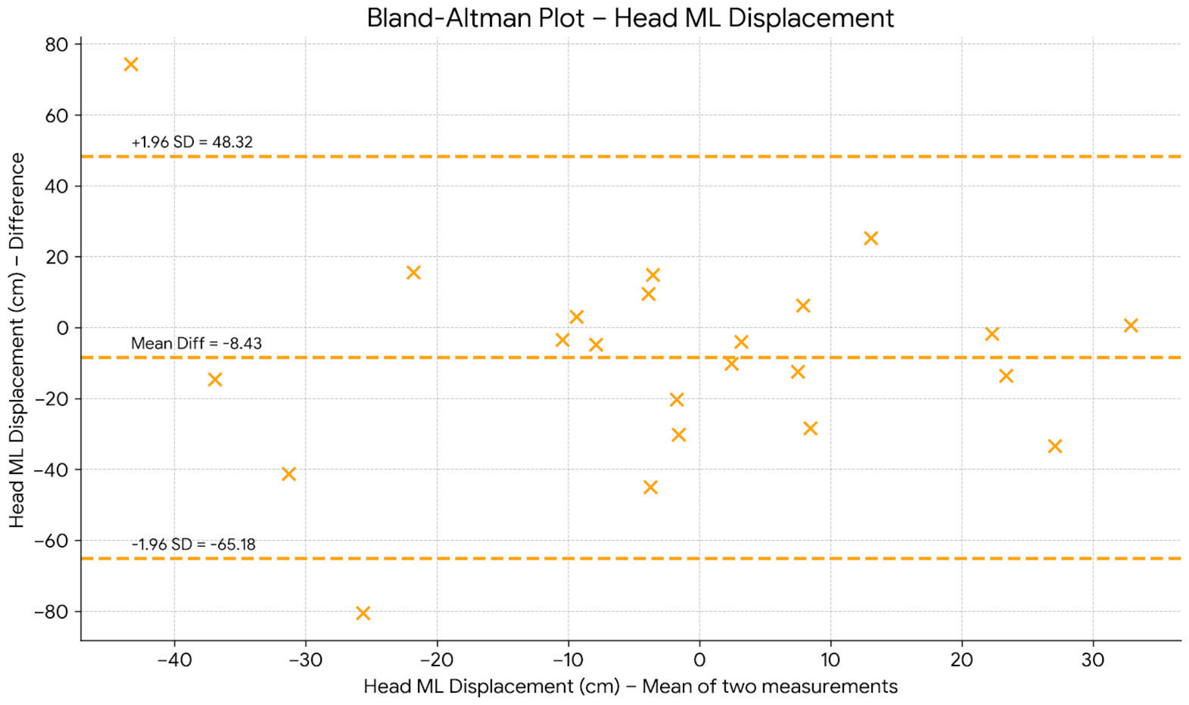

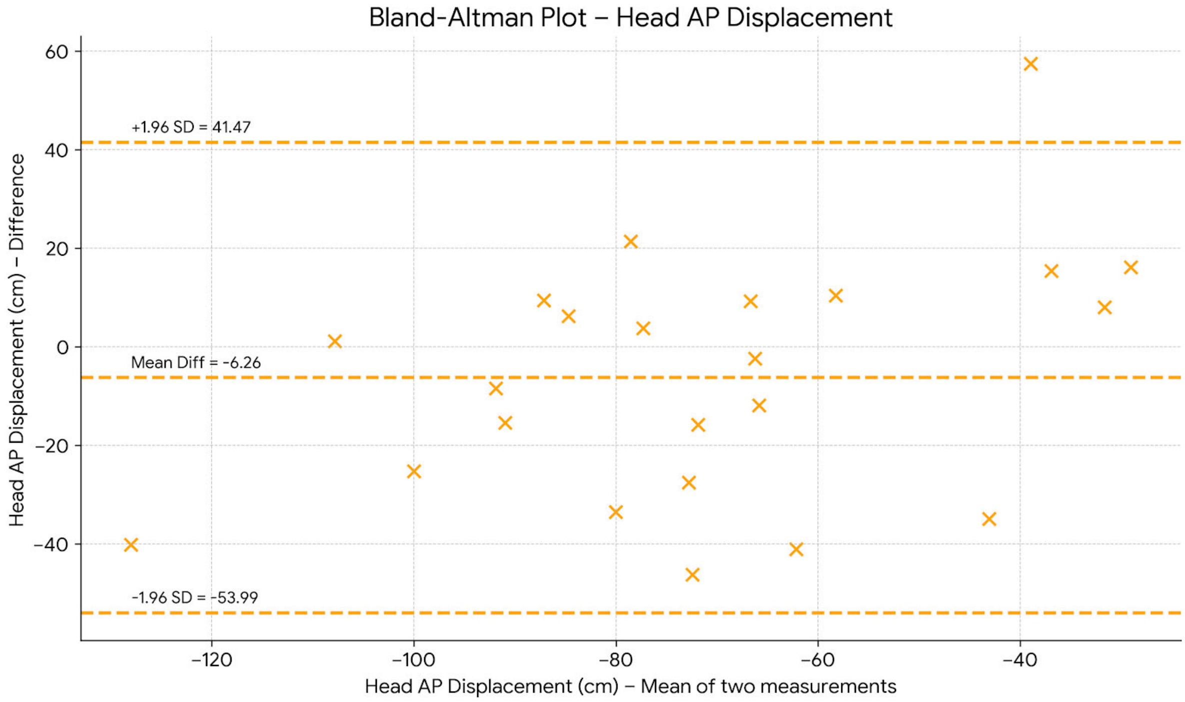
Bland-Altman Plot – Head AP Displacement
Head AP Displacement (cm) – Difference
Head AP Displacement (cm) – Mean of two measurements
+1.96 SD = 41.47
Mean Diff = -6.26
-1.96 SD = -53.99
60
40
20
0
−20
−40
−120
−100
−80
−60
−40

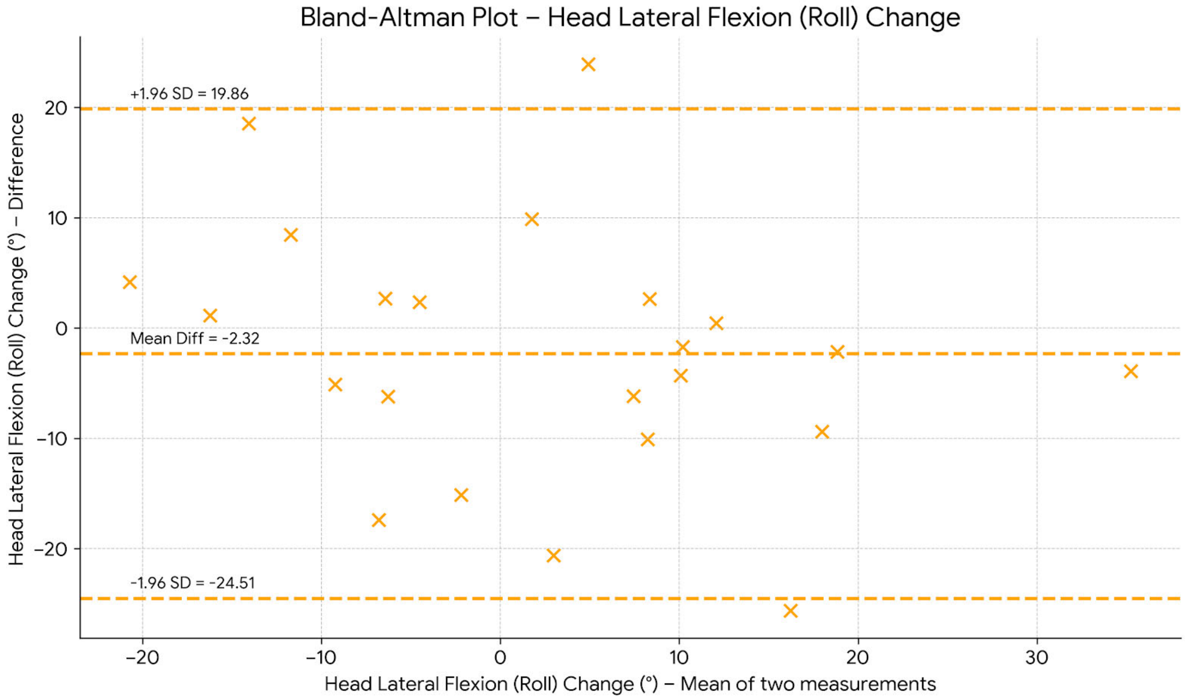
Bland-Altman Plot – Head Lateral Flexion (Roll) Change
Head Lateral Flexion (Roll) Change (°) – Difference
Head Lateral Flexion (Roll) Change (°) – Mean of two measurements
+1.96 SD = 19.86
Mean Diff = -2.32
-1.96 SD = -24.51
20
10
0
−10
−20
−20
−10
0
10
20
30

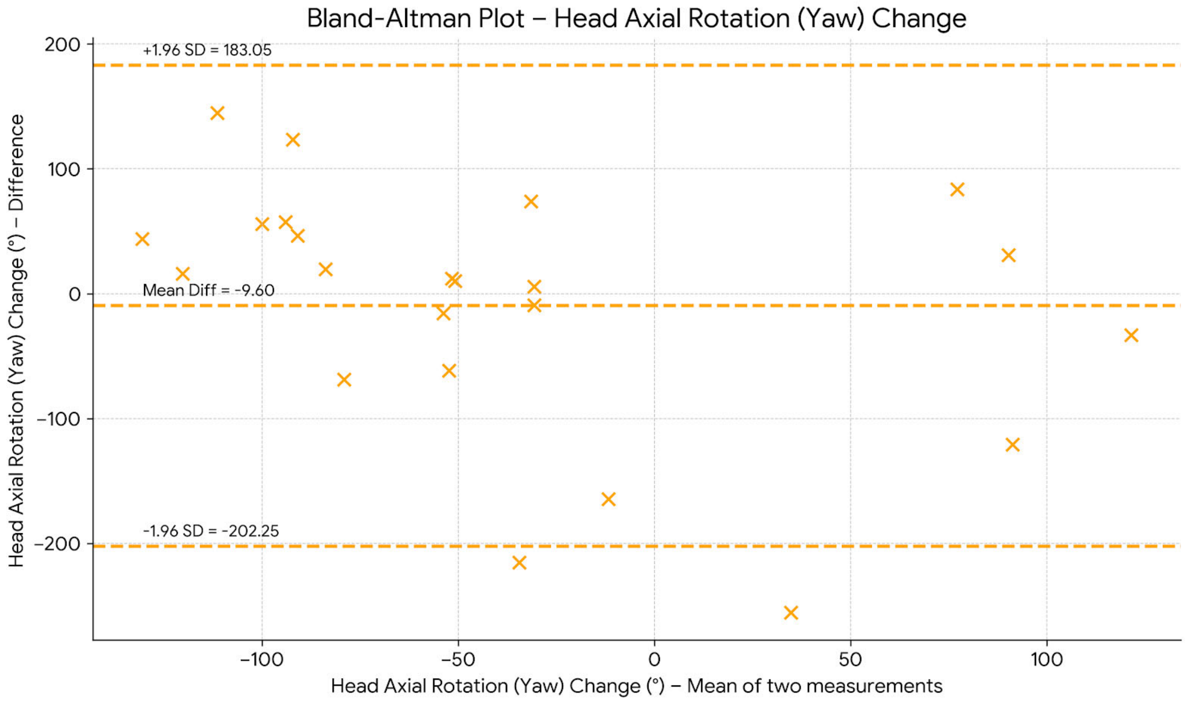
Bland-Altman Plot – Head Axial Rotation (Yaw) Change
+1.96 SD = 183.05
Mean Diff = -9.60
-1.96 SD = -202.25
Head Axial Rotation (Yaw) Change (°) – Difference
Head Axial Rotation (Yaw) Change (°) – Mean of two measurements
200
100
0
−100
−200
−100
−50
0
50
100

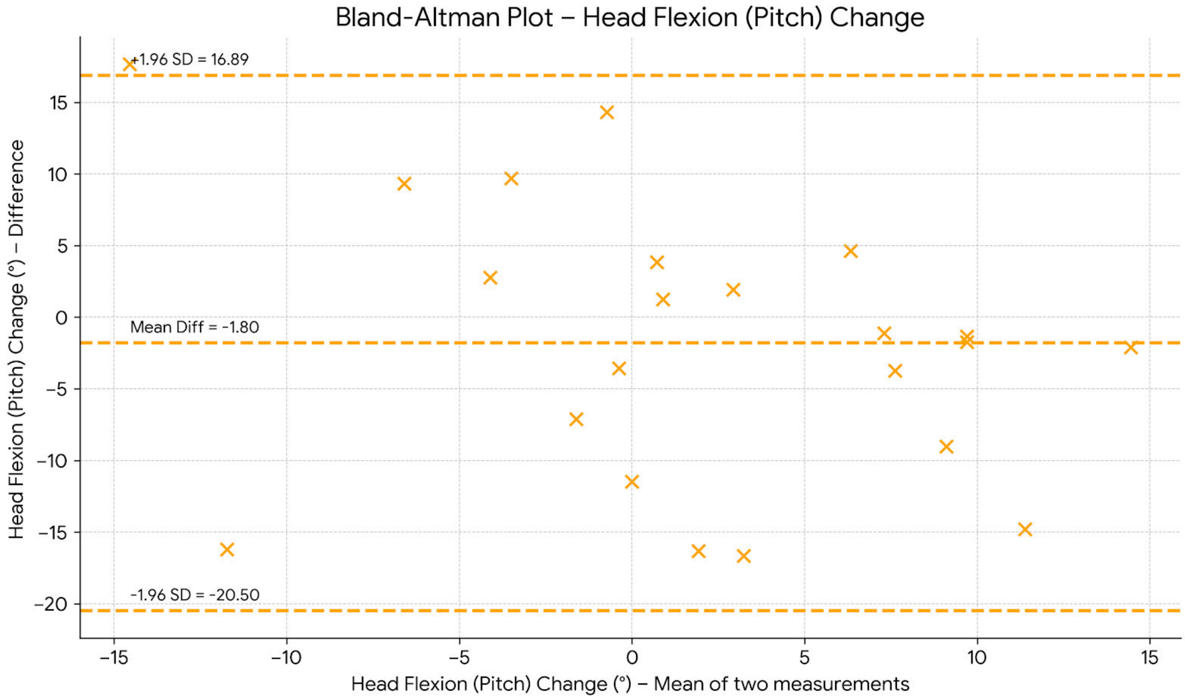
Bland-Altman Plot – Head Flexion (Pitch) Change
+1.96 SD = 16.89
Mean Diff = -1.80
-1.96 SD = -20.50
Head Flexion (Pitch) Change (°) – Difference
Head Flexion (Pitch) Change (°) – Mean of two measurements
15
10
5
0
−5
−10
−15
−20
−15
−10
−5
0
5
10
15

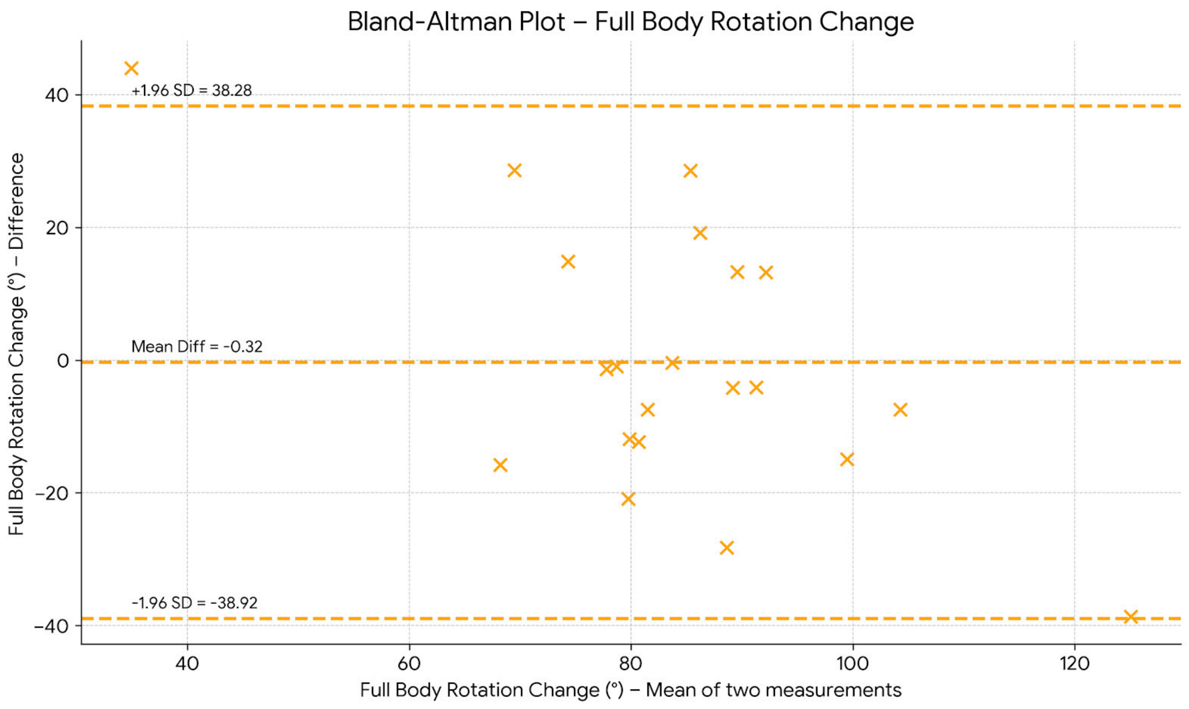
Bland-Altman Plot – Full Body Rotation Change
+1.96 SD = 38.28
Mean Diff = -0.32
-1.96 SD = -38.92
Full Body Rotation Change (°) – Difference
Full Body Rotation Change (°) – Mean of two measurements
40
20
0
−20
−40
40
60
80
100
120

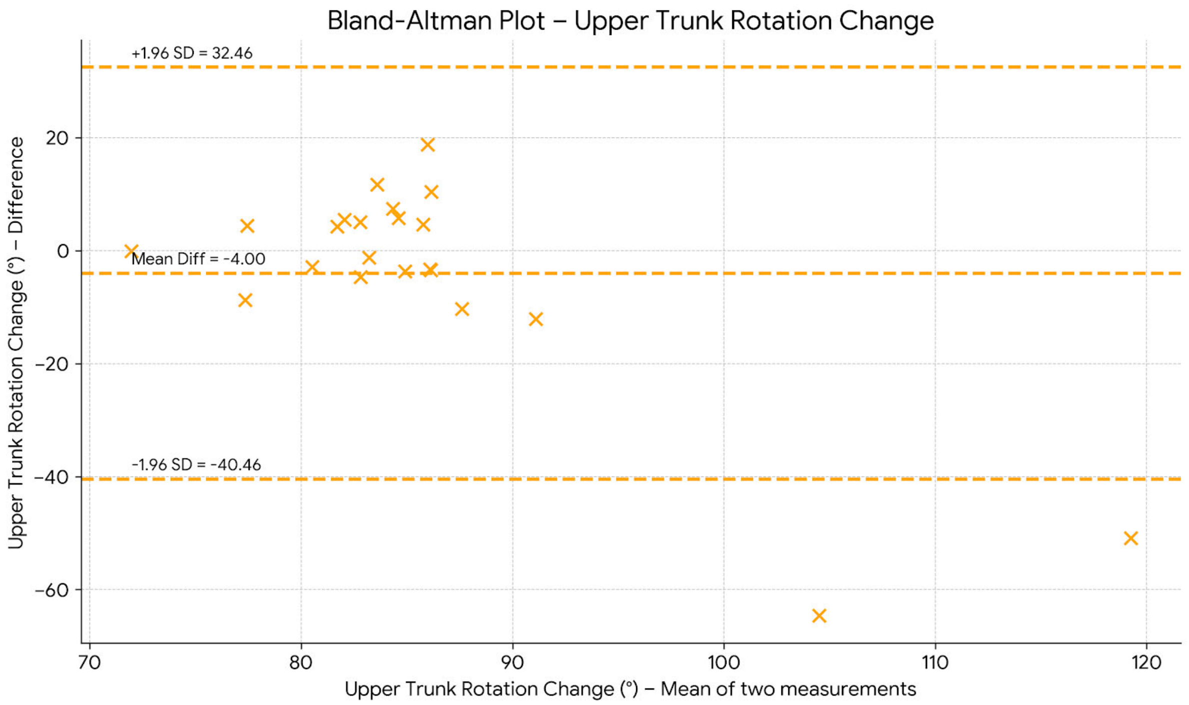
Bland-Altman Plot – Upper Trunk Rotation Change
+1.96 SD = 32.46
Mean Diff = -4.00
-1.96 SD = -40.46
Upper Trunk Rotation Change (°) – Difference
Upper Trunk Rotation Change (°) – Mean of two measurements
20
0
−20
−40
−60
70
80
90
100
110
120

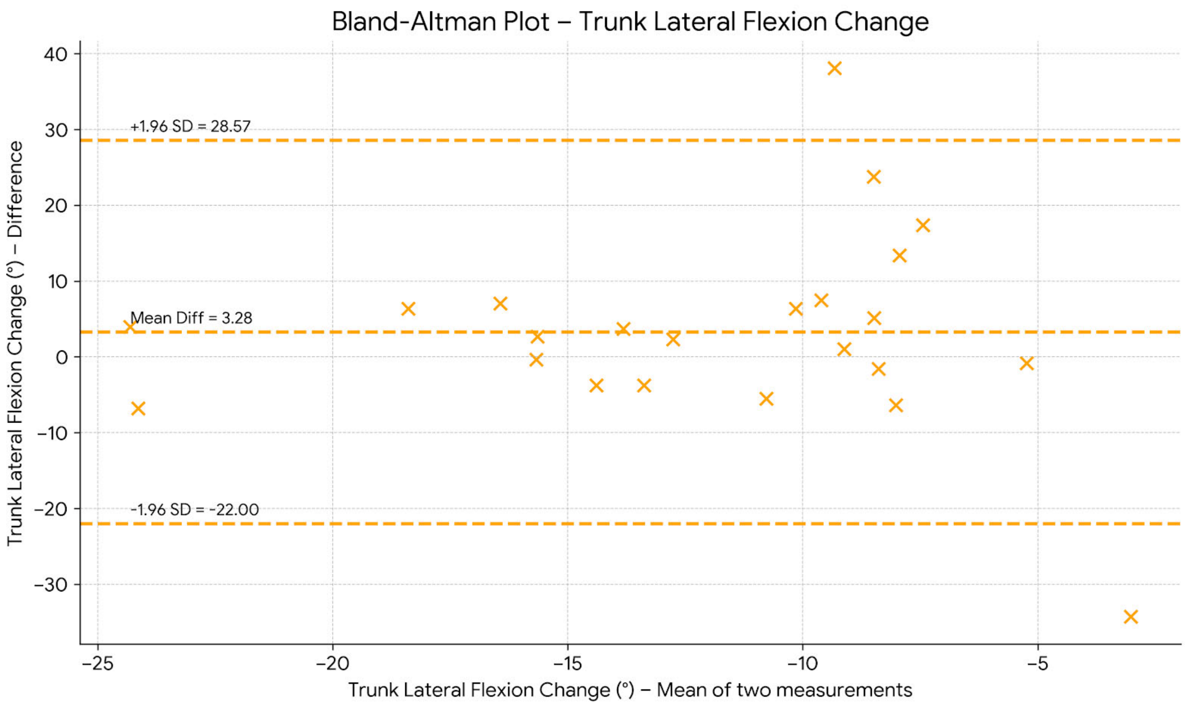

Bland-Altman Plot – Trunk Lateral Flexion Change
+1.96 SD = 28.57
Mean Diff = 3.28
-1.96 SD = -22.00
Trunk Lateral Flexion Change (°) – Difference
Trunk Lateral Flexion Change (°) – Mean of two measurements

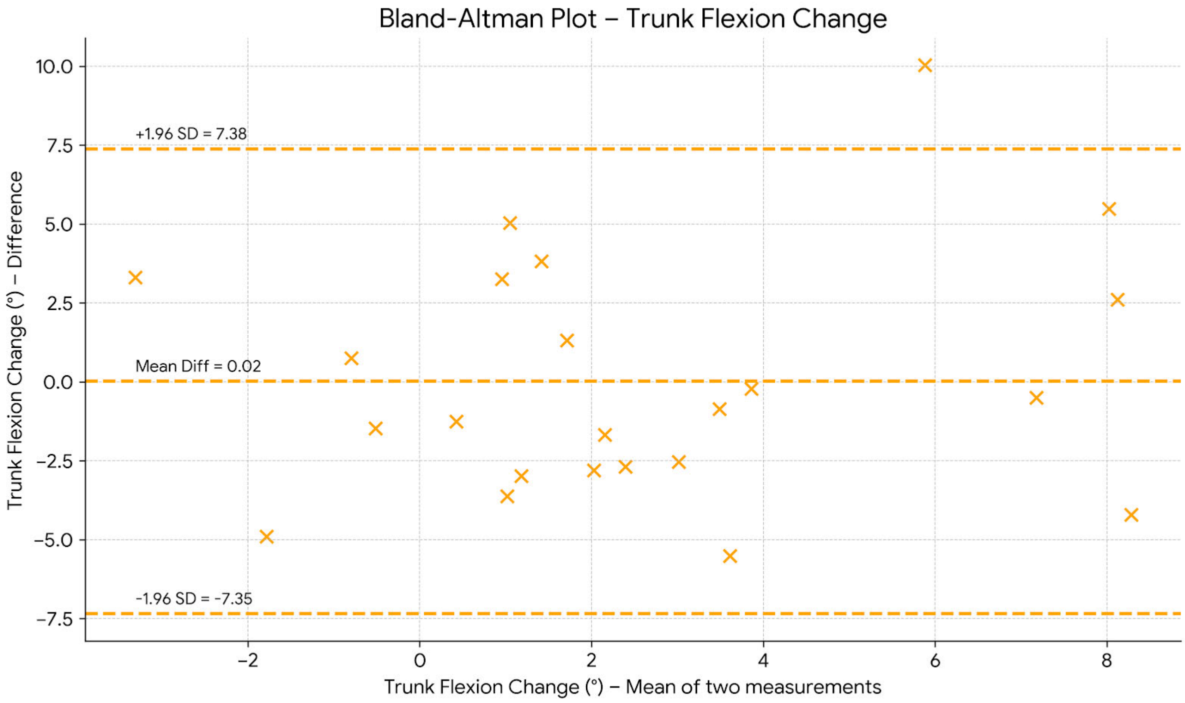

Bland-Altman Plot – Trunk Flexion Change
+1.96 SD = 7.38
Mean Diff = 0.02
-1.96 SD = -7.35
Trunk Flexion Change (°) – Difference
Trunk Flexion Change (°) – Mean of two measurements

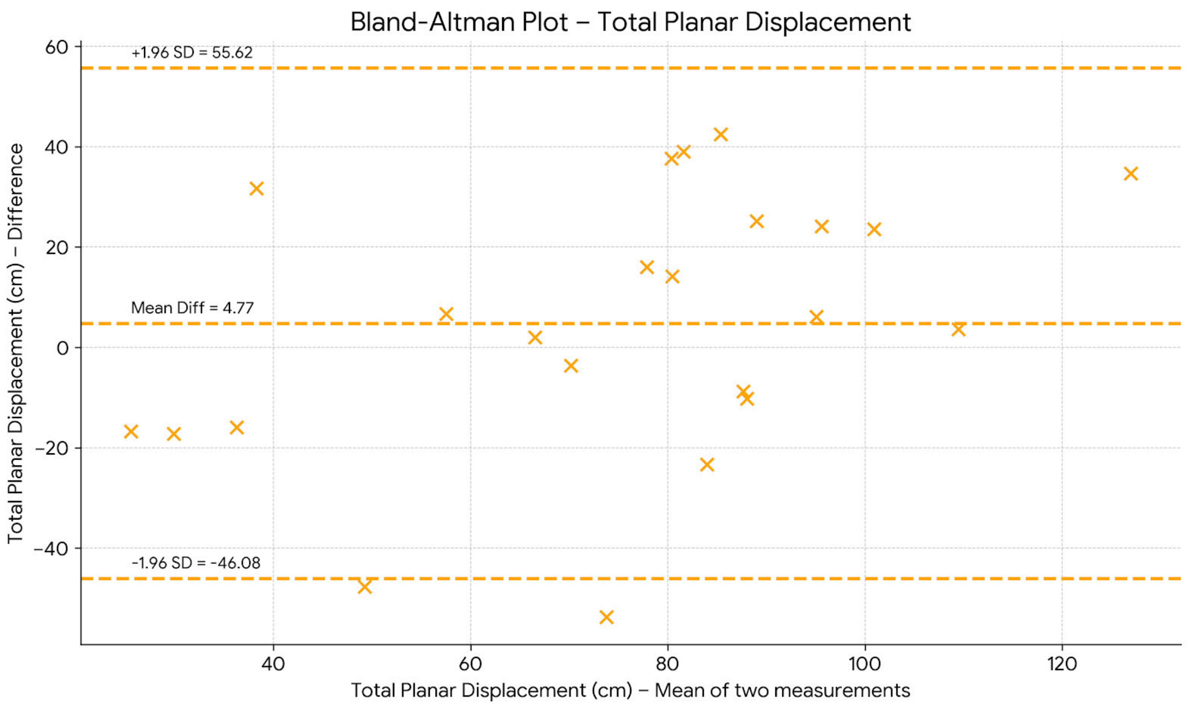
Bland-Altman Plot – Total Planar Displacement
+1.96 SD = 55.62
Mean Diff = 4.77
-1.96 SD = -46.08
Total Planar Displacement (cm) – Difference
Total Planar Displacement (cm) – Mean of two measurements
60
40
20
0
−20
−40
40
60
80
100
120

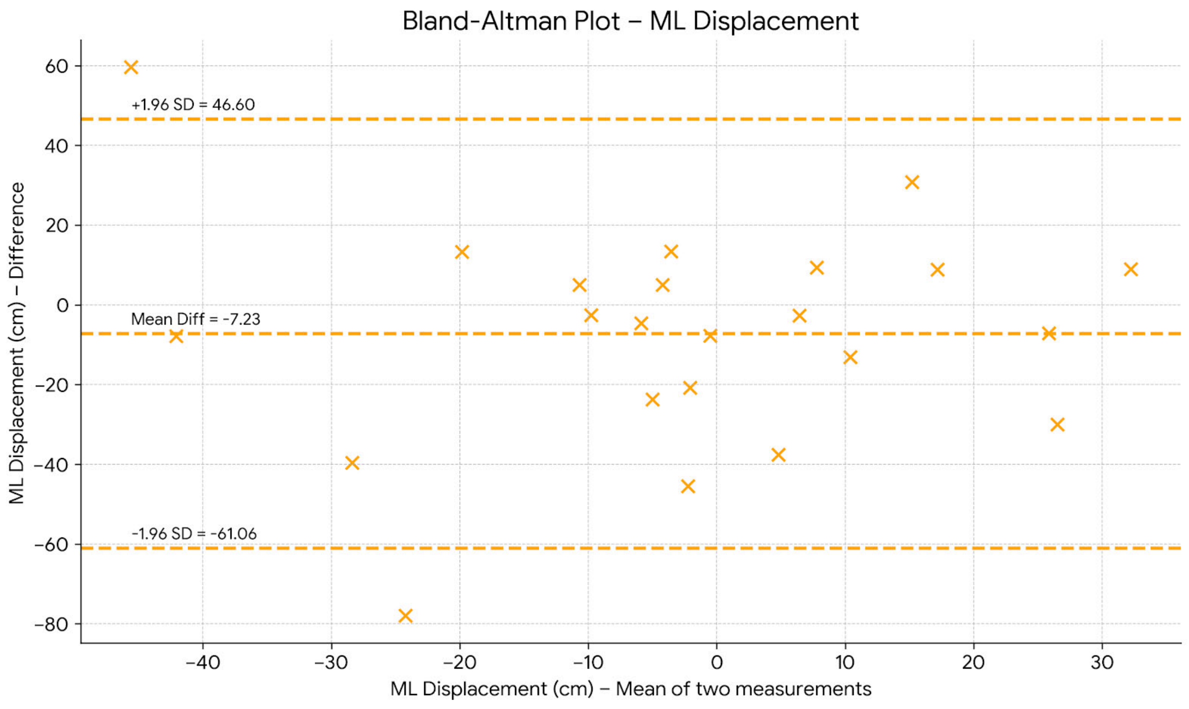
Bland-Altman Plot – ML Displacement
+1.96 SD = 46.60
Mean Diff = -7.23
-1.96 SD = -61.06
ML Displacement (cm) – Difference
ML Displacement (cm) – Mean of two measurements
60
40
20
0
−20
−40
−60
−80
−40
−30
−20
−10
0
10
20
30

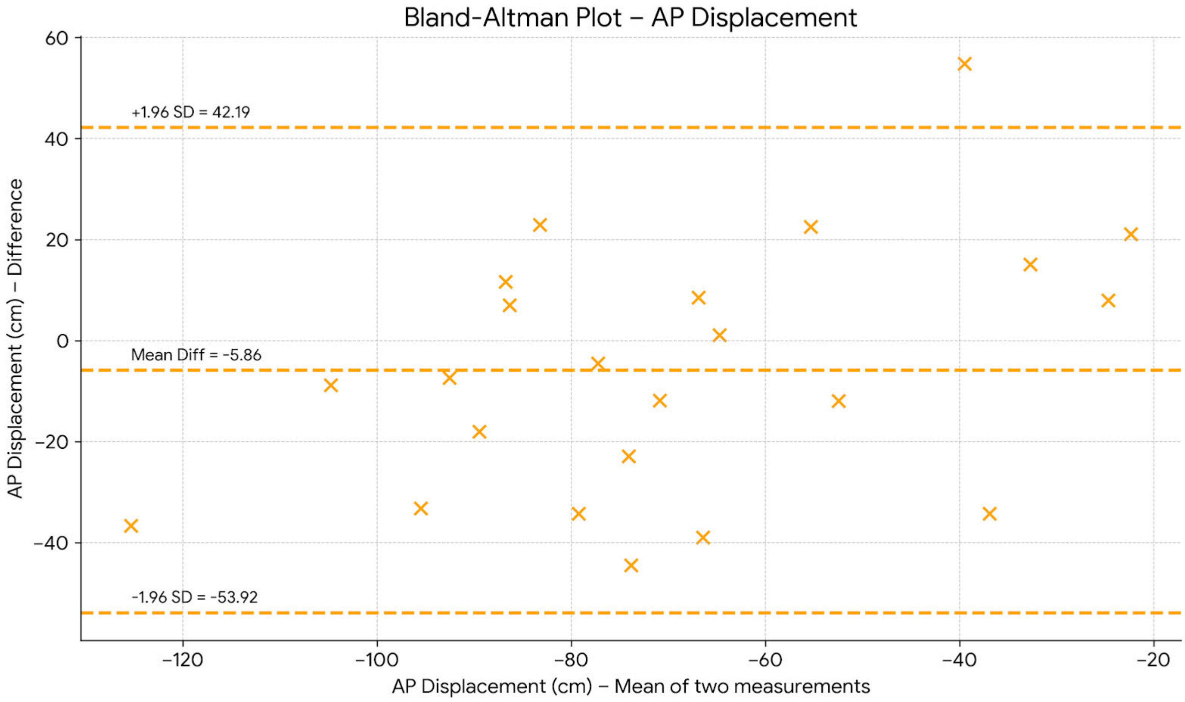

Bland-Altman Plot – AP Displacement
+1.96 SD = 42.19
Mean Diff = -5.86
-1.96 SD = -53.92
AP Displacement (cm) – Difference
AP Displacement (cm) – Mean of two measurements
60
40
20
0
−20
−40
−120
−100
−80
−60
−40
−20
