# Supplementary material for: Depth Sensor-Based Instrumentation of the Fukuda Stepping Test: Reliability and Clinical Associations in Older Adults
Source: Sensors (Basel). 2026 Mar 5;26(5):1623. doi: 10.3390/s26051623 (PMC12986737; doi:10.3390/s26051623)
Supplement: Supplementary file 1 [file sensors-26-01623-s001.zip › Supplementary Material 2 .pdf]

Supplementary Material 2

Data Processing and Kinematic Graphs

Trunk and Body Rotation Calculation

Trunk rotation was quantified using a geometric vector-based approach derived from three-dimensional joint position coordinates. This method directly computes axial rotation from relative segment orientations in the horizontal plane and corresponds to clinically observable postural changes during the Fukuda Stepping Test (FST). All joint coordinates were expressed within a global reference frame defined by the depth sensor system.

Upper Trunk Rotation (Shoulder–Pelvis Rotation)

Upper trunk rotation was calculated as the angular difference between the shoulder girdle and pelvic girdle orientations in the transverse (XZ) plane.

Step 1: Segment Vector Construction

$V\_shoulder(t) = P\_ShoulderRight(t) - P\_ShoulderLeft(t)$

$V\_pelvis(t) = P\_HipRight(t) - P\_HipLeft(t)$

where $P = [X, Y, Z]$ represents the 3D joint position in the global coordinate system.

Step 2: Yaw Angle Calculation

Vectors were projected onto the horizontal plane $V\_h = [X, Z]$, and the yaw angle was calculated using:

$\theta = \mathrm{atan2}(X, Z)$

This yields angles within [−180°, +180°], where positive values indicate rightward rotation.

Step 3: Relative Rotation

$\alpha\_trunk(t) = \theta\_shoulder(t) - \theta\_pelvis(t)$

Step 4: Angle Wrapping

$\alpha\_wrapped(t) = ((\alpha\_trunk(t) + 180°) \bmod 360°) - 180°$

This prevents discontinuities at ±180°.

Step 5: Change from Baseline

$\Delta\alpha\_trunk(t) = \alpha\_wrapped(t) - \alpha\_wrapped(t0)$

where t0 represents the initial time point.

Full-Body Rotation (Shoulder–Ankle Rotation)

Full-body rotation was computed using ankle landmarks instead of hip landmarks:

$V\_base(t) = P\_AnkleRight(t) - P\_AnkleLeft(t)$

$\alpha\_body(t) = \mathrm{wrap180}(\theta\_shoulder(t) - \theta\_base(t))$

Head Axial Rotation

Head axial rotation was calculated relative to shoulder orientation:

$V\_head(t) = P\_Head(t) - P\_Neck(t)$

$\theta\_head(t) = \mathrm{atan2}(X\_head, Z\_head)$

$\alpha\_head(t) = \mathrm{wrap180}(\theta\_head(t) - \theta\_shoulder(t))$

Baseline correction was applied as described above.

Mathematical Summary

$V = P\_right - P\_left$

$\theta = \mathrm{atan2}(X, Z)$

$\alpha = \mathrm{wrap180}(\theta1 - \theta2)$

$\mathrm{wrap180}(\alpha) = ((\alpha + 180°) \bmod 360°) - 180°$

Implementation Details

All calculations were performed in MATLAB R2025a (MathWorks, Natick, MA). The atan2 function was used for quadrant-sensitive angle computation, and angle wrapping was implemented using modular arithmetic.

Limitations

The geometric rotation calculation assumes rigid body segments and does not account for intra-segment deformation. Additionally, the method requires accurate joint tracking by the depth sensor; tracking errors or occlusions may introduce measurement noise.

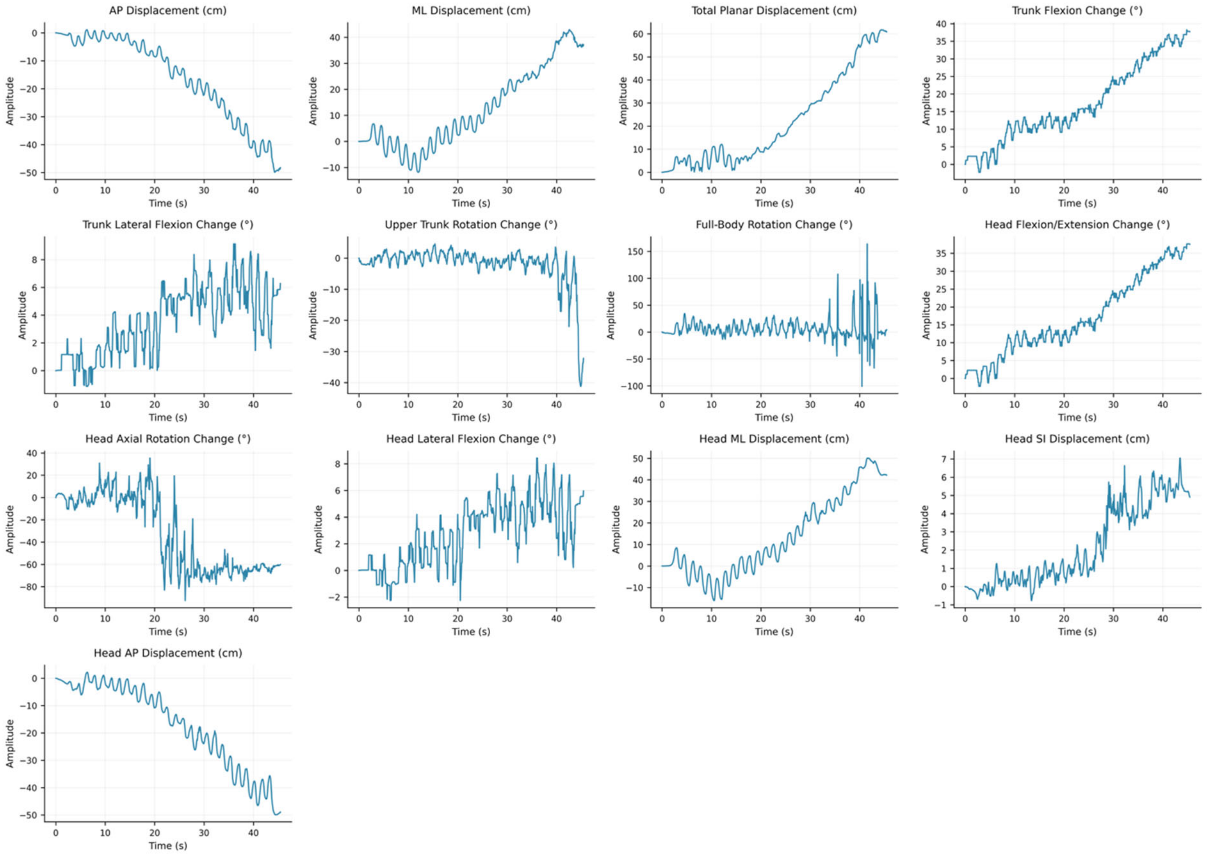

Figure S1. Example visualization of kinematic displacement parameters obtained from a representative participant during the depth sensor–based Fukuda Stepping Test. Trunk anterior–posterior (AP), medio–lateral (ML), and total planar displacements relative to baseline are presented as continuous time-series signals.
